# Inertial-Sensor-Based Monitoring of Sample Entropy and Peak Frequency Changes in Treadmill Walking during Recovery after Total Knee Arthroplasty

**DOI:** 10.3390/s23104968

**Published:** 2023-05-22

**Authors:** Werner A. F. van de Ven, Jurjen Bosga, Wim Hullegie, Wiebe C. Verra, Ruud G. J. Meulenbroek

**Affiliations:** 1 Donders Institute for Brain, Cognition and Behaviour, Radboud University Nijmegen, 6525 GD Nijmegen, The Netherlands; 2 FysioHolland Twente, 7512 AC Enschede, The Netherlands; 3 Physiotherapy Practice Hullegie and Richter MSC, 7512 AC Enschede, The Netherlands; 4 Medisch Spectrum Twente, Department of Orthopedic Surgery, 7512 KZ Enschede, The Netherlands

**Keywords:** entropy, gait, inertial measurement units, knee replacement surgery, power spectral density analysis

## Abstract

This study aimed to investigate whether sample entropy (SEn) and peak frequency values observed in treadmill walking could provide physical therapists valuable insights into gait rehabilitation following total knee arthroplasty (TKA). It was recognized that identifying movement strategies that during rehabilitation are initially adaptive but later start to hamper full recovery is critical to meet the clinical goals and minimize the risk of contralateral TKA. Eleven TKA patients were asked to perform clinical walking tests and a treadmill walking task at four different points in time (pre-TKA, 3, 6, and 12 months post-TKA). Eleven healthy peers served as the reference group. The movements of the legs were digitized with inertial sensors and SEn and peak frequency of the recorded rotational velocity–time functions were analyzed in the sagittal plane. SEn displayed a systematic increase during recovery in TKA patients (*p* < 0.001). Furthermore, lower peak frequency (*p* = 0.01) and sample entropy (*p* = 0.028) were found during recovery for the TKA leg. Movement strategies that initially are adaptive, and later hamper recovery, tend to diminish after 12 months post-TKA. It is concluded that inertial-sensor-based SEn and peak frequency analyses of treadmill walking enrich the assessment of movement rehabilitation after TKA.

## 1. Introduction

### 1.1. Using IMUs in Gait Rehabilitation Following TKA

Assessing the quality of movement behavior during gait rehabilitation is one of the key tasks for physical therapists when monitoring a patient’s recovery after a total knee arthroplasty (TKA). Motion capture systems [1,2,3,4] commonly used in the research of movement behavior in knee osteoarthritis (KO) and after TKA are less applicable in clinical settings due to, for example, a lengthy preparation time for data acquisition, large space requirement to place the cameras, and high additional cost [5]. Inertial measurement units (IMUs), however, provide physical therapists with a solution to overcome the disadvantages of optical motion-capturing systems. IMUs can easily be used in a comfortable way in almost every environment, and they provide a reliable assessment of spatiotemporal gait parameters [5,6,7].

### 1.2. Using Non-Linear Movement Parameters in Gait Rehabilitation Following TKA

Sample entropy (SEn) and peak frequency of the power spectral density function (PSD) have recently been proposed as important measures to capture the dynamics of non-linear systems, which could offer physical therapists with valuable insights into underlying processes determining gait behavior [8]. Harbourne and Stergiou [8] have convincingly argued for the use of non-linear techniques in the assessment of movement behavior in physical therapy practice and in clinical research. Furthermore, it has been demonstrated that non-linear analysis methods can be used to differentiate between TKA and total hip arthroplasty patients during a balancing task [9]. Finally, the use of non-linear motion-analysis techniques during movement rehabilitation could be beneficial for timely recognition of patients with increased risk of overuse injuries due to reduced coordinative variability [10].

### 1.3. Sample Entropy (SEn) and Peak Frequency in Power Spectrum Density (PSD) Analysis

In the present study, SEn was used to assess the predictability of the digitized kinematics of gait movements [11,12]. Low SEn values reveal more similar within-trial fluctuations. This higher-chance occurrence of similar within-trial movement changes reflects, in relative terms, more predictable or stereotypical movement behavior. In contrast, higher SEn values reveal dissimilar within-trial fluctuations reflecting more unpredictable or flexible movement behavior [8]. The second measure analyzed in the present study was the peak frequency of the PSD. With PSD analysis, the power of the different frequencies within a time series are calculated. Although PSD analysis is rarely performed during walking [6,13,14], the scant reports implementing such analysis have proven valuable in studies of walking in KO [6] and in nonspecific lower-back pain [14]. Therefore, PSD analysis might also be promising when assessing the quality of gait movements after TKA.

### 1.4. Aims of Present Study

The primary aim of the present study was to explore whether inertial-sensor-based analyses of SEn and peak frequency could provide a detailed window into gait recovery after TKA. The validation of inertial-sensor-based motion recording and analysis as such was not a specific aim in the present study since earlier studies already showed a good to excellent concurrent validity of inertial-sensor-based motion in the sagittal plane during walking [15,16]. The longitudinal changes in SEn and peak frequency measures of gait kinematics, together with relevant clinical tests capturing gait performance alongside changes, were specifically targeted in the present study.

### 1.5. Empirical Background of the Hypotheses of the Present Study

Various gait characteristics are typical for KO, such as a lower walking speed [2,3,6,17,18,19,20] and lower scores in walking tests including the 6 minute walking test (6 MWT) and timed up-and-go test (TUGT) [21,22]. Patients coping with KO display larger knee stiffness during walking [18], lower variability in the angular velocity of the sagittal knee movements in both the affected and non-affected leg [2,3], and more predictable movement behavior of the shank [23].

TKA is expected to help the patient regain his or her pre-operative walking ability [24,25]. During recovery after a TKA, pre-operative performance levels of the TUGT and 6 MWT have been shown to be accomplished after 3 to 6 months [21,26], but they remained lower than the performance levels of healthy peers [21]. Furthermore, walking speed has been shown to increase after 3 to 12 months post-TKA [2,27], but it remained lower than that of healthy peers [2]. Despite these improved walking abilities, 15% of TKA patients are not satisfied with perceived pain relief during walking after TKA [28]. Although most perceived pain in TKA patients can be explained by prosthetic failure [29,30], some perceived pain in TKA patients remains elusive [29]. Inadequate movement behavior after TKA could play a role in the lack of perceived pain relief after TKA.

Movement strategies that initially are adaptive but later in the rehabilitation process start to hamper recovery can be observed following TKA. Among such movement strategies is, for example, the “stiff knee pattern”, which may be effective at the onset of a knee injury or KO in that it reduces perceived pain and may prevent further injury but becomes persistent when it does not evolve with the changed context, for example, as a result of recurring movement capabilities or in response to the sudden change in sensory consequences associated with TKA [31]. A “stiff knee pattern” strategy, as reflected by a reduced knee excursion and prolonged muscle activation of the tibialis anterior and knee muscles, has been shown to remain present up to 24 months after TKA [1,32]. Decreased variability in sagittal knee movements in both the affected and non-affected leg was still present one year after TKA and has been proved to remain lower when compared to healthy peers [2]. Furthermore, hardly any difference in movement predictability in the sagittal plane of motion during walking was found 6 months after TKA [20]. Finally, despite improved angular velocities of the knee joint during the stance phase 12 to 18 months after TKA, TKA patients show lower angular velocities in the stance phase compared with healthy peers [4].

Aiming to normalize gait, preventing persistent, ineffective movement strategies, and reducing the risk of overuse injuries or a contralateral TKA are key targets in rehabilitation following TKA. People who show a more “stiff knee pattern” gait pattern between 6 and 24 months after a TKA have a greater risk in developing a contralateral TKA even after 6 years [1] and/or overuse injuries due to a reduced coordinative variability [10].

### 1.6. Aims and Predictions

In sum, the aim of the present study was twofold. First, we aimed to gain more insight into gait normalization and persistent movement strategies during treadmill walking after a TKA. Secondly, we aimed to investigate whether inertial-sensor-based gait analysis involving SEn and peak frequency might assist physical therapists to monitor gait recovery after TKA. We expected gait improvement but diminished overall performance one year after a TKA as compared to controls in line with the study of Bade et al. [21]. Furthermore, we expected the amplitude of joint excursion [4] and SEn to increase during rehabilitation. In line with optimal movement variability theory [33], we expected the TKA leg to show lower SEn than the contralateral leg [33]. Finally, we expected larger angular excursion, higher peak frequency, and lower SEn of the IMUs on the shanks as compared to the IMUs on the thighs [34]. Even though we expected that the initially adaptive strategy would diminish during recovery, we did not expect gait behavior to have normalized to the level as displayed by healthy peers after 12 months following TKA.

### 1.7. Main Contributions of the Present Study

- Comparison of clinical gait performance scores obtained from standardized tests to kinematic and dynamic features of treadmill walking during rehabilitation after TKA.
- Identification of a typical “bad habit” adopted in response to knee osteoarthritis and maintained following TKA.
- Evaluation of the use of IMUs and sophisticated dynamic gait analyses as a potential means to enrich the diagnostic and monitoring activities by physical therapists treating TKA patients.

## 2. Materials and Methods

### 2.1. Participants

Eleven participants with unilateral TKA (TKA group) and eleven age- and gender-matched healthy controls (control group) were recruited for this study. The TKA participants were recruited via an orthopedic surgeon and the healthy participants were recruited via social media. The TKA group was measured at four times during the recovery, pre-TKA, ranging from 2 to 19 months pre-TKA (T0), 3 months (T1), 6 months (T2), and 12 months (T3) post-TKA. The control group was measured once and served as a reference for the TKA group. A priori power analysis with a statistical power of 0.8, an α = 0.05, and a η_p_^2^ = 0.07 revealed a total sample size of *n* = 20 [35]. Taking 10% attrition into account, 22 participants, comprising 11 patients and 11 healthy age- and gender-matched controls were included in the study. No differences in age, height, and weight were found between the two groups (Table 1). Participants were included if they met the following inclusion criteria: aged between 50 and 75 years; able to speak, read, and understand Dutch; able to walk without walking aids; no use of orthoses and/or braces for upper and/or lower extremity; and no (history of) neurological of motor diseases and no (history of) severe musculoskeletal injuries of the lower extremity, except the unilateral KO for the TKA group. All participants provided written informed consent for participation in the study. The study was conducted according to the guidelines of the Declaration of Helsinki and was approved by the ethical committee of the Faculty of Social Sciences of the Radboud University, Nijmegen, The Netherlands (ECSW-2019-133) and by the medical ethical committee of Medisch Spectrum Twente, Enschede, The Netherlands (KH20-01).

### 2.2. Clinical Tests

The participants performed the 6 MWT [36,37,38] (walking at comfortable walking speed in a 10 m walkway for six minutes) and the TUGT [36] (rising from an armchair, walking 3 m, turning, returning 3 m, and sitting in the arm chair as fast as possible without running) in order to assess their walking abilities. No IMUs were used during the clinical tests.

### 2.3. Experimental Tasks

Treadmill walking (Excite, Technogym, Cesena, Italy) was used to examine the participants’ movement behavior during walking. During treadmill walking, the participants were supposed to walk without the support of the arms and allowing their arms to swing freely. After the determination of each participant’s individual comfortable walking speed (CWS), a 3 min warm-up at CWS followed in order to get the participants used to the task conditions. IMUs were used during the experimental tasks.

### 2.4. Recording System

**Figure 1:**
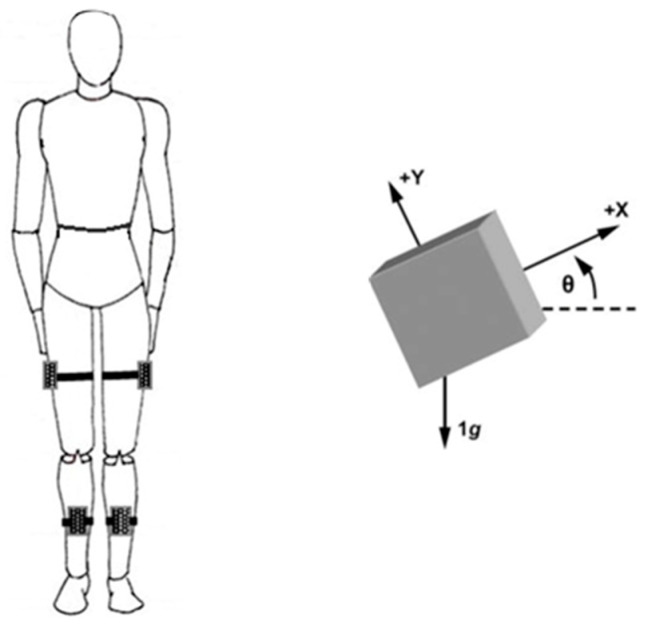
The sensor placement in both the TKA and control groups during treadmill walking (**left**) and depiction of the definition of the amplitude of angular excursion (**right**; see [41]).

Four wireless 3D-inertial motion-capturing sensors (MTw, XSens Technologies B.V., Enschede, The Netherlands) were used to capture the body-segment angular velocity [39], with a 100 Hz sampling rate, of the lower extremities. Following the sensor placement in our previous study [40], the sensors for the thighs were attached midway between the trochanter major and lateral femoral epicondyle on the lateral side of the right and left thigh with Velcro straps (Figure 1). For the shanks, the sensors were attached midway between the apex patella and the malleoli on the anterior side of the right and left shank with Velcro straps (Figure 1). Each sensor sampled in three sensor-based directions (X, Y and Z). SoapSynergy software (v 1.5.1.5, Soapweer B.V., Waalwijk, The Netherlands) was used to record and preprocess the recorded data.

### 2.5. Data Analysis

#### 2.5.1. Data Preprocessing

**Figure 2:**
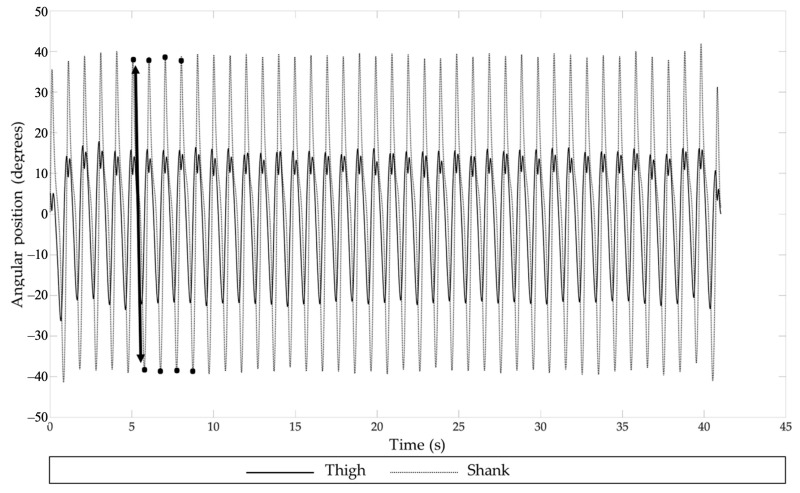
The angular position (degrees) time series of the thigh and shank of one trial performed by a control participant during treadmill walking at CWS. The eight black circles represent a representative subset of the peaks from the shank identified by the peak-to-peak detection algorithm. The black arrow between two successive peaks in the angular position–time function of the shank depicts the angular excursion of the IMU on the shank (due to its fixation around its *Y*-axis) in one cycle ranging between +40 to −40 deg, in a total of 80 degrees. Peak-to-peak detection was visually checked and was subjected to the constraint that positive peak values were always followed by negative peak values, thus eliminating the highly frequent three-peak sequences that can be discerned in the thigh angular position–time function around +10 degrees.

The raw angular velocity signals (deg/s) were preprocessed online with SoapSynergy. First, the raw data were processed with a band-pass third-order Butterworth filter with a cut-off frequency between 0.5 Hz and 20 Hz. Mechanical noise due to oscillations caused by biomechanical structures was minimized by using a 20 Hz cut-off frequency while the interesting higher-movement frequencies were preserved to calculate the SEn [42,43]. The cut-off frequency of 0.5 Hz was used to accommodate potential drift of the data caused by integration during the preprocessing with SoapSynergy. Preprocessing yielded angular position–time functions (degrees; see example in Figure 2) in the X-, Y-, and Z-axes for each sensor separately (see Figure 1).

#### 2.5.2. Kinematics

MATLAB (vR2019b, Mathworks, Natick, MA, USA) was used for subsequent offline processing of the angular position–time functions of the IMUs in order to calculate for each trial the cadence (steps/min) and the mean angular excursion of the realized steps for each sensor in the sagittal plane. For this purpose, the angular position–time signals (degrees) in the sagittal plane (*Z*-axis of the sensors of the thighs and *Y*-axis of the sensors of the shanks) of each IMU were filtered offline in MATLAB with a band-pass third-order Butterworth filter with a bandpass frequency between 0.5 Hz and 6 Hz. The cut-off frequency of 6 Hz was used since a low-pass cut-off frequency of 5×step frequency allows for sufficiently accurate data processing [44]. This filtering technique was applied to facilitate reliable peak-to-peak detection (see also Wang and Ji [45]). To identify movement cycles and determine the cadence and mean amplitude of the angular position–time signal across the steps realized in a trial, a custom-made automatic peak-to-peak detection algorithm was applied (Figure 2). The mean cycle duration (in seconds) for each trial was calculated and converted to the cadence expressed as number of steps per minute. The mean amplitude of angular excursion (degrees) was determined by averaging the amplitudes of the angular excursion of the individual cycles per trial (Figure 2). It must be noted that the latter variable does not represent an anatomical joint angle (e.g., of the hip or knee joint angle) but it represents an angle of the IMU in its local coordinate system given its position in the kinematic chain.

The angular velocity data (deg/s) that had been preprocessed online in SoapSynergy with a bandpass filter of 0.5–20 Hz were used to determine SEn and peak frequency. Preprocessed angular velocity data with an embedding dimension (m) of 2 with a tolerance (r) of 0.2×SD of the angular velocity signal was used for the SEn calculation. The chosen values of the embedding dimension and tolerance are in line with other studies regarding the regularity of cyclical human movement behavior [23,46,47]. First, we designed a template vector with the length of m resulting in:

X_m_(i) = {x_i_, x_i+1_, x_i+2_ …, x_i+m−1_) (1)

Then we calculated the Chebyshev distance, excluding the self-matching case by defining:

d[X_m_(i), X_m_(j)] (i ≠ j) (2)

Thereafter, variables A and B were defined:

A = number of template vector pairs having d[X_m+1_(i), X_m+1_(j)] < r) (3)

B = number of template vector pairs having d[X_m_(i), X_m_(j)] < r) (4)

Finally, SEn was defined by:

SEn = −Ln A/B(5)

To determine the peak frequency, the preprocessed angular velocity data were submitted to spectral density analysis using the Welch power spectral density estimate (MATLAB’s Pwelch method). The peak frequency corresponded with the frequency in the power spectrum with the largest power.

### 2.6. Statistical Analysis

All statistical analyses were performed using IBM SPSS Statistics (version 28.0; IBM Corp., Armonk, NY, USA). The clinical tests (CWS, 6 MWT, and TUGT), cadence, amplitude of angular excursion, peak frequency, and SEn were analyzed in separate repeated-measures analyses of variance (ANOVAs) for the TKA group. The clinical tests and the cadence were evaluated in repeated-measures ANOVA to examine the effect of time (pre-TKA, 3, 6, and 12 months post-TKA). A 2 × 2 × 4 repeated-measures ANOVA was used to examine the effects of TKA (non-TKA vs. TKA leg), segment (thigh vs. shank), and time (pre-TKA, 3, 6, and 12 months post-TKA) on the amplitude of angular excursion, peak frequency, and SEn. The Greenhouse-Geisser correction was used in case of violation of the assumption of sphericity. Polynomial contrasts were used to examine whether the effect of time on clinical tests and kinematics was linear. Because of the violation of normality, Mann-Whitney U tests were used to compare both the clinical tests and kinematics 12 months post-TKA with the control group. A *p*-value of 0.05 or lower was considered statistically significant and *p*-values between 0.05 and 0.10 were considered as a weak trend if the difference in the dependent measure was in the predicted direction.

## 3. Results

Nine participants of the TKA group completed all four measurements (Table 1). One participant was excluded during the study because of a surgical procedure of the non-affected leg and one participant withdrew during the study due to a COVID-19 infection and possible cardiac problems. Eleven healthy peers served as the control group.

### 3.1. Clinical Tests

**Figure 3:**
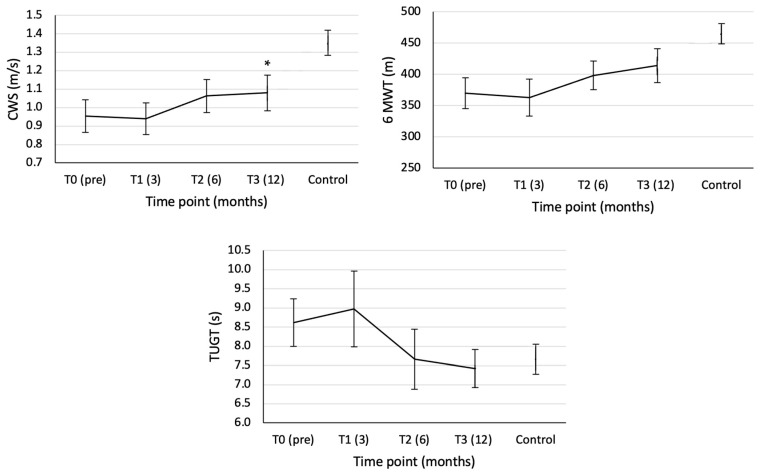
Mean and standard error of the CWS (left upper panel), 6 MWT (right upper panel), and TUG (central lower panel) of the TKA group (*n* = 9) and the control group (*n* = 11). The * indicates a significant difference between the TKA and control group at T3. T0, T1, T2, and T3 represent pre-TKA, 3, 6, and 12 months post-TKA, respectively.

As expected, we found an increased CWS (*p* = 0.021), which showed a trend towards a linear increase (*p* = 0.053) during recovery (Figure 3 and Table 2). However, the CWS 12 months post-TKA remained lower than that of the healthy peers (*p* = 0.025). Furthermore, a linear (*p* = 0.036) increase in the covered distance during the 6 MWT (*p* = 0.007) was found throughout recovery. The performance of the 6 MWT in the TKA group was similar (*p* = 0.230) to that of the control group 12 months post-TKA (Figure 3). The TUGT showed a weak trend to decrease during the rehabilitation after TKA (*p* = 0.093) (Figure 3 and Table 2) confirmed by a weak linear trend (*p* = 0.064) revealed in the polynomial contrasts. The TUGT 12 months post-TKA was comparable with that of the control group (*p* = 0.766) (Figure 3).

### 3.2. Treadmill Walking

#### 3.2.1. Effect of Time

**Figure 4:**
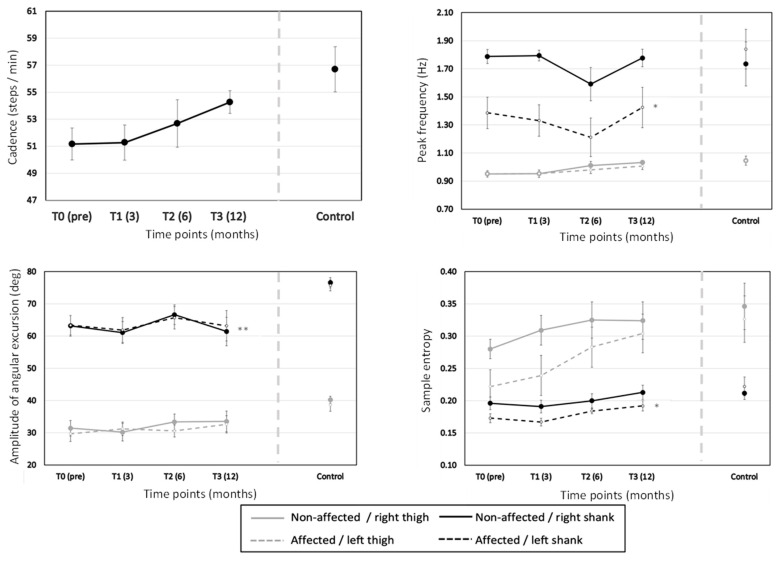
Mean and standard error of the cadence (left upper panel), peak frequency (right upper panel), amplitude of angular excursion (left lower panel), and SEn (right lower panel) of the TKA group (*n* = 9) and control group (*n* = 11). The * indicates a significant difference between the TKA and control group at T3. T0, T1, T2, and T3 represent pre-TKA, 3, 6, and 12 months post-TKA, respectively. Note that the ** indicates a significant difference between the TKA and control group for both the non-affected / right shank and the affected / left shank at T3.

During recovery, a linear trend (*p* = 0.024) but overall non-significant increase in cadence (*p* = 0.203) was found (Table 3 and Figure 4). Furthermore, SEn showed a linear (*p* < 0.001) increase during 12 months after TKA (*p* < 0.001). Finally, no changes in amplitude of angular excursion (*p* = 0.328) and peak frequency (*p* = 0.147) were observed.

##### Effect of TKA

No difference in the amplitude of angular excursion (*p* = 0.742) between the TKA and non-TKA leg was found (Figure 4 and Table 4). In contrast, the TKA leg showed a lower peak frequency (*p* = 0.01) and SEn than the non-TKA leg (*p* = 0.028). The TKA×Time interaction showed that the differences between the TKA and non-TKA leg remained constant for both the peak frequency (*p* = 0.777) and SEn (*p* = 0.403) during 12 months after TKA.

##### Effect of Segment

In line with our hypothesis, the shank showed a larger amplitude of angular excursion (*p* < 0.001), higher peak frequency (*p* < 0.001), and smaller SEn (*p* = 0.002) than the thigh (Figure 4 and Table 5). The Segment×Time interaction revealed that the shanks showed more prominent changes in amplitude of angular excursion (*p* = 0.020) and peak frequency (*p* = 0.037) but a less prominent increase in SEn than the thigh (*p* = 0.024). The absence of Segment×TKA interaction revealed that the TKA had no effect on the differences in cadence (*p* = 0.608), amplitude of angular excursion (*p* = 0.582), and SEn (*p* = 0.177) between the thigh and shank. However, the Segment×TKA interaction (*p* = 0.012) revealed that the TKA leg showed a smaller difference in peak frequency between the thigh and shank than the non-TKA leg. The Segment×TKA×Time interaction (*p* = 0.473) revealed that the TKA effect for the peak frequency did not change throughout 12 months after TKA.

##### TKA vs. Control Group

When compared with the control group, the TKA group 12 months post-TKA showed a weak trend towards a lower cadence, a lower amplitude of angular excursion for both shanks, a lower peak frequency of the TKA shank, and a lower SEn of the TKA shank (Figure 4 and Table 6).

## 4. Discussion

In the present study, we found, as expected, an improved walking performance 12 months post-TKA. Furthermore, in line with our hypotheses, we found that restrictive gait strategies, in particular an adaptive gait pattern characterized by a stiffened knee, gradually diminished in the TKA group during recovery. The expected differences 12 months post-TKA compared with the control group were also confirmed. Finally, the results showed that SEn and peak frequency measures indeed reflect movement quality during walking after TKA, as expected, and were therefore proven to be suitable to monitor gait recovery after TKA in clinical settings.

### 4.1. Recovery after TKA

The observed improvement in walking performance in the TKA group, reflected by a higher CWS after 6 and 12 months, improvement in the 6 MWT distance after 6 and 12 months, and faster time on the TUGT after 12 months, is in line with previous studies [2,21,26,27]. Furthermore, the lower CWS of the TKA group 12 months after TKA compared with healthy peers is also in line with a previous study [2]. However, the comparable performance of the 6 MWT and TUGT between the TKA group with healthy peers 12 months after TKA is in contrast with a previous study [21]. The use of different time points in the comparison between the TKA and control group (6 months in Bade et al. [21] vs. 12 months in the present study) could explain these differences.

Despite the improved walking performance, we found, in line with our hypotheses, persisting adaptations in movement behavior throughout the recovery after TKA. Regarding the amplitude of angular excursion of the legs, we unexpectedly found no increase in amplitude of angular excursion during recovery after TKA. After a TKA, the knee joint is potentially less constrained due to osteophytes and swelling which potentially restricts the range-of-motion in the case of KO [48]. We presume that the perpetuated smaller angular excursion could be attributed to persisted adaptation [31], probably caused by prolonged muscle activation around the knee [32]. Since knee osteoarthritis is a slow progressive musculoskeletal disorder [48], gait behavior will, in principle, adapt to structural changes in the joint to ensure walking ability. Theoretically, after a TKA, adaptive gait behavior is also expected to change over time. However, a “stiff knee pattern” adaptive gait behavior, reflected by a smaller knee excursion [1], probably caused by prolonged muscular co-contractions around the knee [32], still lingers after 12 months of recovery time. This explanation suggests that the neuromotor system requires more than 12 months to adapt adequately to the consequences of a TKA, which is strengthened by the fact that the shanks showed unsystematic changes in amplitude of angular excursion during the recovery. Walking speed is determined by step length and frequency [49]. The fact that our results showed increased CWS but no increase in the amplitude of angular excursion can explain the observed linear increased cadence 12 months after TKA.

Regarding the peak frequency, we found a lower peak frequency and a smaller difference between the thigh and shank in the TKA leg, which both increased throughout recovery. The found lower peak frequency was mainly caused by the lower peak frequency of the TKA shank. The differences in the peak frequency between the TKA and non-TKA shank reflect, in our view, the degree to which the legs exploit the higher harmonic frequency [50] because of the leg impacting the ground and inducing reaction force main peaks at twice the gait frequency. Exploiting the ground reaction forces is subserved by a full extension of the knee joint during the weight bearing phase. This process is hampered because the TKA leg lacks the coordination dynamics inducing full knee joint extension. To nevertheless accommodate weight bearing, the TKA shank takes part in a typical “stiffer knee gait” as a consequence of a co-contraction strategy [1,32]. This kind of movement behavior is undesirable since people with an increased “stiff legged” gait pattern who have undergone a TKA have a greater risk in developing a contralateral TKA within 6 years [1].

In line with our expectations, we found SEn increasing during recovery, indicating less predictable movement behavior of the legs 6 and 12 months post-TKA. Our findings are in contrast with the findings of Roelofsen et al. [20], who hardly found any differences in SEn 18–20 weeks post-TKA. An explanation for the differences in results could be related to the usage of the CWS after TKA. Roelofsen et al. [20] used the same CWS pre-TKA and post-TKA, whereas in our study the CWS was determined at every time point in the recovery. Six months post-TKA, we found an increased CWS and previous studies showed that higher walking speeds elicited larger SEn [23,51,52].

Despite the increased SEn, the TKA leg showed lower SEn, and therewith more predictable movement behavior, than the non-TKA leg throughout the recovery after TKA. This finding is in line with the optimal movement variability theory which states that musculoskeletal disorders such as KO and recovery after TKA show more regular and predictable movement behavior [33]. The lower SEn of the TKA leg could have clinical implications since a reduced coordinative variability is associated with overuse injuries [10]. After a TKA, 5–10% of the patients experience anterior knee pain [53] and perceived anterior knee pain can be seen a result of “overloading” [53,54]. TKA patients who were able to adapt and reduce joint loading experienced less or no anterior knee pain [54], and after a 6-week gait-retraining program, runners showed increased approximate entropy values of the kinematics and kinetics that are known to reflect variations in patellofemoral stress [55]. In extension of these studies, we presume that the more predictable movement behavior of the TKA leg could lead to overloading and therefore a higher risk of perceived anterior knee pain.

Finally, the observed larger SEn of the thighs compared to the shank is understandable and corroborates the leading joint hypothesis [34]. During walking, the movement of the leg at the beginning of the swing phase is initiated at the hip and thigh, and results in a larger interaction torque at the knee joint and shank and a higher angular velocity of the knee joint [56] and shank [57]. During walking, the leg swing can predominantly be a ballistic movement which will lead to lower variability in the shank due to inertia exploitation. This presumption is strengthened by the fact that variability is lower in the direction of progression in comparison to the orthogonal directions [58]. The fact that the differences in SEn between the thighs and shanks became more prominent during the recovery after TKA suggests that the TKA group was able to exploit the inertia during the swing phase of both legs more than pre-TKA.

### 4.2. Health and Clinical Implications

Despite the observed modulations in gait behavior during the 12-month post-TKA period, the gait behavior of TKA patients after 12 months was still not normal as compared to healthy adults. Several other studies showed comparable differences in walking abilities and movement behavior during walking between TKA and healthy adults [2,4,21,27,32]. We presume that the persisting adaptive gait behavior with which the TKA group is still coping after 12 months can be termed a “bad habit” [31]. This “bad habit” could be attributed to the compensatory strategy that the patient developed pre-TKA in response to KO and raises an interesting question whether the length of an admission process for a TKA is a deciding factor that codetermines the development of a “bad habit” [31] or whether the neuromotor system requires more than 12 months to overcome this “bad habit”. Since expectations prior to a TKA influence the satisfaction rate after TKA [59], we suggest that the development of “bad habits” and the minimal time required to overcome these bad habits should be part of patient information prior to the TKA.

Furthermore, the persistent differences in peak frequency and SEn between the TKA and non-TKA leg suggest that patients after TKA are at risk for developing overuse injuries of the TKA leg and higher risk of a contralateral TKA. This presumes that the movement quality plays a role in the recovery after TKA and should be addressed in the management of both KO and TKA. However, the current guidelines of KO and TKA lack the assessment of movement quality during gait [48,60], and clinical tests, such as 6 MWT, fail to assess the movement quality.

With respect to the “bad habits” discussed above, it is relevant to note that at the level of muscular activity, the differential, excessive co-contraction of proximal musculature supporting balance and posture may also play an important role [2,32,61].

The present study shows that inertial sensors and non-linear analysis are valuable tools for characterizing movement quality changes during the recovery after TKA, in accordance with previous studies [6,7,8]. In addition, given the fact that inertial sensors can easily be used in a comfortable way in almost every environment [5], they are helpful in monitoring recovery after TKA in clinical settings, such as physical therapy. Furthermore, the SEn and peak frequency afford important insights into both the movement quality during walking and the recovery of the neuromotor system, and more so than merely cadence and amplitude measures. In particular, the use of SEn and peak frequency provides clinicians with means to identify and recognize initially adequate adaptive behavior that lingers unconstrained and therefore potentially hampers further recovery after TKA. This is crucial during rehabilitation after a TKA in order to help the TKA patients to meet their expectations regarding their walking abilities [24,25] and to reduce the risk for a contralateral TKA and overuse injuries [1,10].

### 4.3. Limitations and Further Study

A limitation of this study is the number of TKA participants who completed all the measurements. The power of the study was affected because two participants of the TKA group dropped out. However, despite the two dropouts, we have shown that initially persisting adaptive gait behavior that hampers recovery was diminished 12 months post-TKA, but never regained to the level of healthy controls. These findings must, nevertheless, be interpreted with some caution.

Another limitation was the long timespan of the pre-TKA measurements. Due to the COVID-19 pandemic, surgical treatments were postponed. Some participants of the TKA group were thus forced to compensate longer for their arthritic knee and this could have led to a more serious “bad habit”.

Finally, since the use of inertial sensors combined with non-linear analysis techniques seems valuable for clinical settings, future studies could provide more insights into the extension of these applications outside clinical settings. Nowadays, mobile apps are used to provide more insight for a clinician into the activity of TKA patients during their daily living [62,63], and adding information about the quality of movement during daily activities could give the clinician a more complete view of the recovery process of the TKA patient.

## 5. Conclusions

An improved walking performance and diminished limiting gait adaptations were found 12 months after TKA. However, differences in movement behavior in gait were still present compared to healthy peers. The present study demonstrates that inertial sensors and SEn and peak frequency are valuable tools and measures for characterizing movement quality during treadmill walking after TKA. This methodology is therefore suitable for monitoring the recovery after TKA in clinical settings.

## Figures and Tables

**Table 1:** Demographics (mean (M) and standard deviation (SD)) of the included participants of the TKA group and the control group. Note that differences between the TKA group (T0) and (T3) are caused by exclusion of two TKA participants during the evaluations.

	TKA Group (T0) (5 Males; 6 Females) (M ± SD)	TKA Group (T3) (4 Males; 5 Females) (M ± SD)	Control Group (5 Males; 6 Females) (M ± SD)	*p*-Value T0 vs. Control	*p*-Value T3 vs. Control
Age (years)	64.00 ± 5.00	64.33 ± 5.39	61.00 ± 4.67	0.161	0.155
Height (meters)	1.74 ± 0.11	1.74 ± 0.11	1.73 ± 0.09	0.832	0.828
Mass (kilograms)	94.00 ± 16.96	95.00 ± 16.36	81.91 ± 16.92	0.110	0.098
Comorbidities	None	None	None		
Number of evaluations	1	4	1		

**Table 2:** Mean (M), standard error (SE), and test statistics of the repeated-measures ANOVA with polynomial contrasts of the changes during recovery for the mean CWS, 6 MWT, and TUGT. T0, T1, T2, and T3 represent pre-TKA, 3, 6, and 12 months post-TKA, respectively, for the TKA group only (*n* = 9). No data of IMUs were used.

Clinical Tests	Time Point (Months)				Test Statistics	Polynomial Contrasts
	T0 (pre)	T1 (3)	T2 (6)	T3 (12)		
	M	M	M	M		
	(SE)	(SE)	(SE)	(SE)		
CWS (m/s)	0.954 (0.089)	0.940 (0.086)	1.063 (0.090)	1.080 (0.097)	F(3,24) = 3.889, *p* = 0.021, η_p_^2^ = 0.327	Linear: F(1,8) = 5.131, *p* = 0.053, η_p_^2^ = 0.391
6 MWT (m)	369.66 (24.63)	362.89 (29.67)	398.28 (23.01)	414.06 (27.20)	F(3,24) = 5.126, *p* = 0.007, η_p_^2^ = 0.391	Linear: F(1,8) = 6.299, *p* = 0.036, η_p_^2^ = 0.441
TUGT (s)	8.61 (0.62)	8.97 (0.99)	7.66 (0.78)	7.42 (0.50)	F(3,24) = 2.394, *p* = 0.093, η_p_^2^ = 0.230	Linear: F(1,8) = 4.631, *p* = 0.064, η_p_^2^ = 0.367

**Table 3:** Mean (M), standard error (SE) per time point of both the TKA and non-TKA leg, and test statistics of the repeated-measures ANOVA with polynomial contrasts of the changes during time for the cadence, amplitude of angular excursion, Sen, and peak frequency. T0, T1, T2, and T3 represent pre-TKA, 3, 6, and 12 months post-TKA, respectively, for the TKA group only (*n* = 9). Analysis was limited to the sagittal plane, and the statistics resulting from the processing of the Z-data of the IMU on the thigh and of the Y-data of the IMU on the shank for each time point were pooled across 4 IMUs.

Parameters	Time Point (Months)				Test Statistics	Polynomial Contrasts
	T0 (pre)	T1 (3)	T2 (6)	T3 (12)		
	M	M	M	M		
	(SE)	(SE)	(SE)	(SE)		
Cadence (steps per min)	51.0 (1.20)	51.0 (1.20)	52.8 (1.80)	54.6 (0.60)	F(3,24) = 1.658, *p* = 0.203, η_p_^2^ = 0.172	Linear: F(1,8) = 7.774, *p* = 0.024, η_p_^2^ = 0.493
Amplitude of angular excursion (degrees)	46.91 (2.50)	46.10 (2.88)	49.05 (2.55)	47.69 (3.53)	F(3,24) = 1.207, *p* = 0.328, η_p_^2^ = 0.131	Linear: F(1,8) = 0.572, *p* = 0.471, η_p_^2^ = 0.067
Sen	0.22 (0.01)	0.23 (0.01)	0.25 (0.01)	0.26 (0.01)	F(3,24) = 8.476, *p* < 0.001, η_p_^2^ = 0.514	Linear: F(1,8) = 40.394, *p* < 0.001, η_p_^2^ = 0.835
Peak frequency (Hz)	1.27 (0.04)	1.26 (0.03)	1.20 (0.06)	1.31 (0.04)	F(3,24) = 1.960, *p* = 0.147, η_p_^2^ = 0.197	Linear: F(1,8) = 0.288, *p* = 0.606, η_p_^2^ = 0.035

**Table 4:** Mean (M), standard error (SE) of the TKA and non-TKA leg, and test statistics of the repeated-measures ANOVA for the effects of TKA and TKA×Time for the amplitude of angular excursion, SEn, and peak frequency for the TKA group only (*n* = 9). Analysis was limited to the sagittal plane. The statistics resulting from the processing of the Z-data of the IMU on the thigh and of the Y-data of the IMU on the shank were pooled for each leg separately.

Parameters	TKA Leg	Non-TKA Leg	Test Statistics
	M	M	
	(SE)	(SE)	
Amplitude of angular excursion (degrees)	47.27 (3.00)	47.60 (2.49)	TKA: F(1,8) = 0.117, *p* = 0.742, η_p_^2^ = 0.014 TKA×Time: F(3,24) = 2.482, *p* = 0.085, η_p_^2^ = 0.237
SEn	0.22 (0.01)	0.26 (0.02)	TKA: F(1,8) = 7.173, *p* = 0.028, η_p_^2^ = 0.473 TKA×Time: F(3,24) = 1.016, *p* = 0.403, η_p_^2^ = 0.113
Peak Frequency (Hz)	1.16 (0.06)	1.36 (0.03)	TKA: F(1,8) = 11.356, *p* = 0.010, η_p_^2^ = 0.587 TKA×Time: F(3,12.207) = 0.184, *p* = 0.777, η_p_^2^ = 0.022

**Table 5:** Mean (M), standard error (SE) of the thigh and shank, and test statistics of the repeated-measures ANOVA for the effects of segment, Segment×Time, Segment×TKA, and Segment×TKA×Time for the amplitude of angular excursion, SEn, and peak frequency for the TKA group only (*n* = 9). Analysis was limited to the sagittal plane. The statistics resulting from the processing of the Z-data of the IMU on the thigh and of the X-data of the IMU on the shank were pooled for the thighs and shanks separately.

Parameters	Thigh	Shank	Test Statistics
	M	M	
	(SE)	(SE)	
Amplitude of angular excursion (degrees)	31.58 (2.14)	63.29 (3.34)	Segment: F(1, 8) = 521.344, *p* < 0.001, η_p_^2^ = 0.985 Segment×Time: F(3,24) = 3.938, *p* = 0.020, η_p_^2^ = 0.330 Segment×TKA: F(1,8) = 0.329, *p* = 0.582, η_p_^2^ = 0.040 Segment×TKA×Time: F(3,24) = 0.785, *p* = 0.514, η_p_^2^ = 0.089
SEn	0.29 (0.02)	0.19 (0.01)	Segment: F(1,8) = 20.743, *p* = 0.002, η_p_^2^ = 0.722 Segment×Time: F(3,24) = 3.781, *p* = 0.024, η_p_^2^ = 0.321 Segment×TKA: F(1,8) = 2.188, *p* = 0.177, η_p_^2^ = 0.215 Segment×TKA×Time: F(3,24) = 1.342, *p* = 0.284, η_p_^2^ = 0.144
Peak Frequency (Hz)	0.98 (0.01)	1.54 (0.06)	Segment: F(1,8) = 91.086, *p* < 0.001, η_p_^2^ = 0.919 Segment×Time: F(3,24) = 3.317, *p* = 0.037, η_p_^2^ = 0.293 Segment×TKA: F(1,8) = 10.565, *p* = 0.012, η_p_^2^ = 0.569 Segment×TKA×Time: F(3,24) = 0.865, *p* = 0.473, η_p_^2^ = 0.098

**Table 6:** Mean (M) and standard error (SE) for the amplitude of angular excursion, SEn, and peak frequency per segment per time point (T0, T1, T2, T3) for the TKA group, and for the control group. T0, T1, T2, and T3 represent pre-TKA, 3, 6, and 12 months post-TKA, respectively. Data shown in Figure 4.

		Parameters		
		Amplitude of Angular	SEn	Peak Frequency (Hz)
Time Points	Segment	Excursion (Degrees)		
(Months)		M (SE)	M (SE)	M (SE)
T0 (pre)	Non-TKA thigh	31.43 (2.34)	0.28 (0.03)	0.95 (0.02)
	Non-TKA shank	63.16 (3.20)	0.20 (0.01)	1.79 (0.05)
	TKA thigh	29.66 (2.41)	0.22 (0.02)	0.95 (0.02)
	TKA shank	63.37 (3.02)	0.17 (0.01)	1.39 (0.11)
T1 (3)	Non-TKA thigh	30.19 (2.09)	0.31 (0.03)	0.95 (0.03)
	Non-TKA shank	61.08 (3.42)	0.19 (0.01)	1.79 (0.04)
	TKA thigh	31.24 (2.76)	0.24 (0.02)	0.95 (0.03)
	TKA shank	61.85 (3.87)	0.17 (0.01)	1.33 (0.11)
T2 (6)	Non-TKA thigh	33.37 (1.86)	0.33 (0.03)	1.01 (0.03)
	Non-TKA shank	66.63 (3.05)	0.20 (0.01)	1.59 (0.12)
	TKA thigh	30.56 (2.49)	0.28 (0.03)	0.98 (0.03)
	TKA shank	65.66 (3.48)	0.18 (0.004)	1.21 (0.14)
T3 (12)	Non-TKA thigh	33.53 (2.67)	0.32 (0.03)	1.03 (0.02)
	Non-TKA shank	61.42 (4.41)	0.21 (0.01)	1.78 (0.06)
	TKA thigh	32.64 (3.19)	0.30 (0.03)	1.01 (0.01)
	TKA shank	63.16 (4.73)	0.19 (0.01)	1.43 (0.14)
Controls	Right thigh	40.21 (2.01)	0.35 (0.04)	1.05 (0.03)
	Right shank	76.56 (1.58)	0.21 (0.01)	1.74 (0.16)
	Left thigh	38.69 (1.10)	0.33 (0.04)	1.05 (0.03)
	Left shank	75.72 (1.70)	0.22 (0.02)	1.84 (0.14)

## Data Availability

The data presented in this study are available on request from the corresponding author. The data are not publicly available yet due to problems in processing the request for a publicly available data collection.

## Glossary

TKA	total knee arthroplasty
KO	knee osteoarthritis
SEn	sample entropy
PSD	power spectral density
6 MWT	6 minute walking test
TUGT	timed up-and-go test
CWS	comfortable walking speed
